# Effects of Zinc Acetate on Serum Zinc Concentrations in Chronic Liver Diseases: a Multicenter, Double-Blind, Randomized, Placebo-Controlled Trial and a Dose Adjustment Trial

**DOI:** 10.1007/s12011-019-01851-y

**Published:** 2019-08-07

**Authors:** Kazuhiro Katayama, Atsushi Hosui, Yoshiyuki Sakai, Minoru Itou, Yasushi Matsuzaki, Yoriyuki Takamori, Keiko Hosho, Tomomi Tsuru, Yasuhiro Takikawa, Kojiro Michitaka, Eishin Ogawa, Yoko Miyoshi, Toshifumi Ito, Shinobu Ida, Izumi Hamada, Katsunori Miyoshi, Hiroko Kodama, Tetsuo Takehara

**Affiliations:** 1 Department of Hepato-biliary and Pancreatic Oncology, Osaka International Cancer Institute, 3-1-69, Otemae, Chuo-ku, Osaka, 541-8567 Japan; 2 Department of Gastroenterology and Hepatology, Osaka Rosai Hospital, Osaka, Japan; 3 Division of Hepatobiliary and Pancreatic Diseases, Department of Internal Medicine, Hyogo College of Medicine, Nishinomiya, Hyogo Japan; 4 Department of Internal Medicine, Kurume Clinical Pharmacology Clinic, Fukuoka, Japan; 5 Department of Gastroenterology and Hepatology, Tokyo Medical University Ibaraki Medical Center, Ibaraki, Japan; 6 Department of Internal Medicine, Teikyo University Hospital, Tokyo, Japan; 7 2nd Department of Internal Medicine, Tottori University Hospital, Tottori, Japan; 8 Department of Rheumatology, PS Clinic, Fukuoka, Japan; 9 Division of Hepatology, Department of Internal Medicine, Iwate Medical University Hospital, Iwate, Japan; 10 Department of Gastroenterology, Ehime Prefectural Central Hospital, Matsuyama, Ehime Japan; 11 Department of Pediatrics, Teikyo University Hospital, Tokyo, Japan; 12 Department of Pediatrics, Osaka University Graduate School of Medicine, Osaka, Japan; 13 Department of Gastroenterology and Hepatology, Japan Community Healthcare Organization Osaka Hospital, Osaka, Japan; 14 Department of Pediatric Gastroenterology and Nutrition, Osaka Women’s and Children’s Hospital, Osaka, Japan; 15 Department of Research and Development, Nobelpharma Co., Ltd., Tokyo, Japan; 16 Department of Health and Dietetics, Faculty of Health and Medical Science, Teikyo Heisei University, Tokyo, Japan; 17 Department of Gastroenterology and Hepatology, Osaka University Graduate School of Medicine, Osaka, Japan

**Keywords:** NPC-02, Zinc deficiency, Hypozincemia, Chronic liver disease, Dose adjustment, Serum zinc concentration

## Abstract

The essential trace element zinc maintains liver functions. Liver diseases can alter overall zinc concentrations, and hypozincemia is associated with various hepatic pathologies. Modulating systemic zinc through dietary supplementation is potentially useful for liver diseases. We evaluated the usefulness of zinc (NPC-02; acetate formulation) supplementation. We conducted two NPC-02 studies on zinc-deficient patients (serum zinc < 70 μg/dL). Study 1: double-blind, randomized, placebo-controlled trial on 57 subjects with chronic liver diseases comparing serum zinc in patients given NPC-02 (NPC-02 group) versus placebo (Placebo group). Study 2: dose adjustment study on 43 subjects with/without liver diseases to determine proportions maintaining serum zinc target (≥ 80 μg/dL but < 200 μg/dL). In study 1, NPC-02 subjects had higher serum zinc concentrations at week 8 than Placebo subjects (83.2 ± 20.2 and 61.3 ± 12.0, respectively; *P* < 0.0001), and more NPC-02 than Placebo subjects achieved the serum zinc target (15/27 vs. 1/26). In study 2, the NPC-02-induced serum zinc increase was dose-dependent in subjects both with and without liver diseases (*r* = 0.5143, *P* = 0.0022 and *r* = 0.5753, *P* = 0.0005, respectively). Interestingly, there was a marginally positive correlation between serum zinc and albumin levels in subjects with but not in those without liver diseases (*r* = 0.4028, *P* = 0.0631 and *r* = 0.1360, *P* = 0.5567, respectively). NPC-02 dose-dependently increases serum zinc in hypozincemic patients, regardless of liver disease. NPC-02 is a potentially effective therapy for liver cirrhosis, in which zinc deficiency is common. Clinical trial registry number: NCT02337569, NCT02321865.

**Electronic supplementary material:**

The online version of this article (10.1007/s12011-019-01851-y) contains supplementary material, which is available to authorized users.

## Introduction

Zinc, an essential trace element, is involved in the enzymatic activities and structural maintenance of numerous enzymes and proteins, and it has various physiological roles in the body. Specifically, zinc works as a growth factor and exerts immunomodulatory, antioxidant, anti-apoptotic, and anti-inflammatory effects [1–3]. Accordingly, zinc plays important roles in many biological phenomena, and therefore its homeostasis is maintained systemically. The amount of zinc in the body is adjusted via absorption in the digestive tract and excretion into feces; therefore, zinc deficiency can be caused by a long-term decrease in zinc uptake [2, 3]. Zinc deficiency is also observed in various diseases (e.g., liver cirrhosis, chronic renal diseases, and inflammatory bowel disease), contributing to the underlying pathological conditions [3–8]. Notably, zinc is necessary for maintenance of normal liver function, and the liver plays an important role in maintenance of zinc homeostasis. Thus, the systemic zinc concentration is influenced by liver diseases, and zinc deficiency is involved in the pathological processes of liver diseases [2, 3]. Further, Hosui et al. recently showed that the level of serum zinc concentration could predict hepatocarcinogenesis in patients with liver cirrhosis who had been treated with zinc supplementation [9]. Therefore, the serum level of zinc concentration is an important factor in clinical settings.

This study examined the effects of administering a zinc acetate formulation on the serum zinc concentration along with related blood test results in patients with liver diseases. Many previous studies also focused on liver diseases and zinc, but most examined the relationships between the serum zinc concentration and the pathological conditions of liver diseases [2–4, 10–13]. Despite the amount of zinc in the serum accounting for only approximately 0.1% of total zinc in the body, the serum zinc concentration and the pathologies of specific diseases were found to show correlations in many studies [3]. Additionally, zinc administration-induced alterations in the serum zinc concentration and changes in disease pathology also showed correlations. In an animal study, the serum zinc concentrations and those in hepatic tissue showed correlations [14]. Therefore, the serum zinc concentration may be a useful index for assessing pathological conditions and evaluating treatments for liver diseases. Furthermore, determining how to adjust systemic serum zinc concentrations through dietary supplementation may provide information useful for the management of liver diseases. Herein, we report two trials of the administration of a zinc acetate formulation, NPC-02, to patients with hypozincemia.

## Materials and Methods

Two separate multicenter studies of the zinc acetate formulation NPC-02 (25 mg of Zn/tablet; Nobelpharma Co., Ltd., Tokyo, Japan) were conducted.

### Study 1: a Double-Blind, Randomized, Placebo-Controlled Trial of NPC-02 in Patients with Zinc Deficiency

The purpose of this double-blind, randomized, placebo-controlled trial (RCT) was to evaluate the efficacy (increase in serum zinc concentration) and safety of NPC-02.

The primary endpoint was serum zinc concentration changes at week 8, and the secondary endpoints were the proportions of subjects whose serum zinc concentration had reached at least 80 μg/dL at weeks 4 and 8 in the NPC-02 and Placebo groups.

Based on the results of a previous clinical study [13], we assumed that a change in the active drug group (receiving 50 mg/day) would be 20 μg/dL while that in the placebo group would be 0 μg/dL. When the standard deviation for both groups was set at 20 μg/dL, the two-sided significance level (*α*) set to 0.05, and the statistical power (1 − *β*) set at 0.9, an appropriate sample size was calculated to be 23 patients per group (1:1). Taking possible dropouts into account, the target sample size was set at 25 patients per group, with a total of 50 for the two groups.

From January to September 2015, 57 outpatients were assessed for eligibility to participate in Study 1 at nine sites. The inclusion criteria were as follows: (1) ≥ 20 years of age, (2) serum zinc concentration of < 70 μg/dL (hypozincemia was defined as < 80 μg/dL; however, taking dietary changes into account, it was set at < 70 μg/dL). The exclusion criteria were (1) severe hepatitis, (2) malignancy, (3) severe heart, blood, kidney, or pancreatic disease, (4) blood albumin concentration of < 2.8 g/dL, (5) allergy to formulations containing zinc, (6) consumption of a zinc formulation in the previous 12 weeks, (7) pregnant or potentially pregnant, (8) participation in other clinical studies in the previous 12 weeks, or (9) judged ineligible for this study by the investigators.

The study discontinuation criteria were as follows: (1) request by subjects or guardians to withdraw from the study, (2) judged to have difficulty continuing the study due to worsening of primary diseases or complications, (3) serum copper concentration < 10 μg/dL, (4) inclusion criteria not applicable or confirmed to conflict with the exclusion criteria, (5) concomitant use of prohibited medications, (6) changing hospitals, (7) failure to return for hospital visits, (8) pregnancy confirmed, (9) other reasons including investigator’s (or sub-investigator’s) judgment that a subject should be withdrawn from the study.

All 57 subjects assessed for eligibility were confirmed to be eligible for Study 1, and were thus enrolled in the study. Of these, 31 subjects were randomly allocated to the NPC-02 group, and 26 were allocated to the Placebo group based on a preset randomized code. Randomization was performed using the block randomization method (block size, 6) by a scientist not involved in the study to ensure blinding of researchers. The codes were kept secure until decoding after completion of the data analysis.

NPC-02 (containing 25 mg of zinc) and the placebo were administered twice daily for 8 weeks in the NPC-02 and Placebo groups, respectively. Registered patients, except those who had not been administered NPC-02 and those for whom no information had been obtained on efficacy after the start of administration, were included in the full analysis set (FAS).

The study was registered with Clinical Trials.gov (NCT 02337569).

### Study 2: Dose Adjustment Study of NPC-02 in Patients with Zinc Deficiency

The purpose of this study was to identify the optimal dose adjustment method for NPC-02 administration to achieve the target serum concentration (≥ 80 μg/dL but < 200 μg/dL) in patients with hypozincemia, and to evaluate the maintenance effect of NPC-02 administration once the target serum concentration has been reached. The maximum amount of zinc was set at 150 mg/day. This study was conducted in two groups: (1) adult patients with chronic liver diseases, and (2) adult patients without liver diseases as well as pediatric patients free of hepatic disorders but with hypozincemia.

From January to July 2015, 43 patients who met the criteria described below were enrolled at 10 sites (22 adult patients who had been diagnosed as having chronic liver diseases, and 21 patients without chronic liver diseases including 9 children). The inclusion criteria were (1) serum zinc concentration of < 70 μg/dL and (2) ability to take the study drug. The patient exclusion criteria and the study discontinuation criteria were the same as those specified for Study 1. In addition, Study 2 was discontinued for the subjects who had difficulty with dose adjustments. All 43 subjects were included in the FAS analysis.

The serum zinc concentration was measured every 4 weeks. When it was below the target concentration (≥ 80 μg/dL but < 200 μg/dL), the NPC-02 dose was increased. The maximum amount of zinc was set at 150 mg/day for subjects with a body weight of ≥ 30 kg and 75 mg/day for those weighing < 30 kg, with an administration frequency of 3 times/day. Once the target concentration was reached, the therapy was further continued at the same dose and completed after 8 weeks of maintenance NPC-02 administration. In addition, when the concentration exceeded the target range (200 μg/dL), the dose was decreased by 25 mg/day.

This study was registered with Clinical Trials.gov (NCT 02321865).

### Ethics

The study protocols conformed to the ethical guidelines of the 1975 Declaration of Helsinki as reflected in the prior approval granted by the institutional review board of each institution including Osaka International Cancer Institute (Project identification code: Study 1, A-2015-01-0216, and Study 2, A-2015-01-0215; Date of approval, January 8, 2015; Name of the institutional review board, Institutional Review Board of Osaka Medical Center for Cancer and Cardiovascular Diseases; The name of hospital was changed to Osaka International Cancer Institute in March 2017). The details of this clinical trial were fully explained to each patient in both oral and written form, and written consent was obtained from each patient.

### Statistical Analyses

Study 1 (RCT): For the primary efficacy endpoint, the differences between serum zinc concentration changes in the NPC-02 and Placebo groups were compared by applying analysis of covariance with respective baseline values as covariates. For the secondary endpoint, the difference between the groups in the proportions of subjects whose serum zinc concentration was ≥ 80 μg/dL, and its 95% confidence interval were calculated. For comparisons of background characteristics between the two groups, Fisher’s exact probability test was used for categorical variables, and the two-sample *t* test for continuous variables. The Mann-Whitney test was used for analyzing changes from baseline in clinical laboratory parameters, for comparisons between the two groups.

All statistical analyses were performed at a significance level of 0.05.

Study 2 (dose adjustment study): The proportions of subjects who maintained the target serum zinc concentration (≥ 80 μg/dL but < 200 μg/dL) were evaluated. For comparison of correlations between the serum zinc concentration and the NPC-02 dose, as well as between the baseline blood and albumin concentrations of the two groups, linear regression analysis and the correlation coefficients were used. For comparison of the two groups in terms of presence/absence of liver diseases, Fisher’s exact probability test was used for analyzing categorical variables, and the two-sample *t* test for continuous variables.

## Results

### Study 1: a Double-Blind RCT of NPC-02 in Patients with Zinc Deficiency

The baseline characteristics of subjects included in this study are listed in Table 1. There were no significant differences between the NPC-01 and Placebo groups, except in body weight, which was greater in the NPC-02 group.

**Table 1:** Baseline characteristics of subjects in Study 1

Parameter	NPC-02 group (*n* = 31)	Placebo group (*n* = 26)	*P* value
Sex (M/F)	17/14	10/16	0.289^a^
Age (years)	64.2 ± 12.5	67.8 ± 12.2	0.279^b^
Body weight (kg)	65.34 ± 14.49	55.59 ± 13.22	0.004^b*^
Primary disease	Chronic hepatitis/liver cirrhosis/hepatic dysfunction	Chronic hepatitis/liver cirrhosis/hepatic dysfunction	–
	10/17/4	13/13/0	
	Causes of liver disease (HBV/HCV/HBV + HCV/Alcohol/ NAFL/PBC/NBNC/others)	Causes of liver disease (HBV/HCV/HBV + HCV/Alcohol/ NAFL/PBC/NBNC/others)	
	1/15/1/6/0/0/6/2	1/17/0/2/0/1/5/0	
ALT (U/L)	31.5 ± 14.2	47.0 ± 39.6	0.369^b^
AST (U/L)	44.4 ± 19.2	51.1 ± 27.4	0.437^b^
ALP (U/L)	427.7 ± 264.0	467.3 ± 329.0	0.660^b^
Total bilirubin (mg/dL)	1.09 ± 0.66	0.84 ± 0.35	0.221^b^
Albumin (g/dL)	3.65 ± 0.46	3.79 ± 0.50	0.372^b^
Amylase (U/L)	93.3 ± 28.6	103.5 ± 37.8	0.614^b^
Lipase (U/L)	54.9 ± 21.3	57.2 ± 24.6	0.841^b^
Creatinine (mg/dL)	0.763 ± 0.218	0.685 ± 0.205	0.192^b^
Zinc (μg/dL)	58.6 ± 13.2	60.1 ± 9.9	0.659^b^

Values are expressed as means ± SD

*ALP* alkaline phosphatase, *ALT* alanine aminotransferase, *AST* aspartate aminotransferase, *HBV* hepatitis B virus, *HCV* hepatitis C virus, *NAFL* nonalcoholic fatty liver, *NBNC* non-hepatitis B virus and non-hepatitis C virus, *PBC* primary biliary cholangitis

*P < 0.05 was considered statistically significant

^a^Test used for analysis: Fisher’s exact probability test

^b^Test used for analysis: two-sample *t* test

**Fig. 1:**
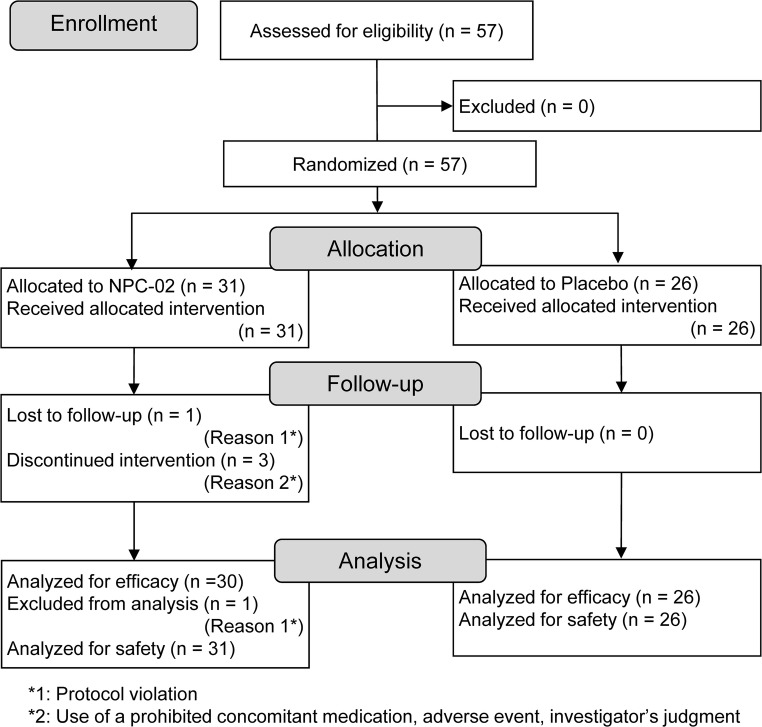
Flow diagram of subjects in Study 1

In the NPC-02 group, one subject was excluded from the efficacy analysis due to the use of a prohibited concomitant medication (the subject took the study drug for only 3 days, and thus lacked data for the efficacy analysis), and three subjects discontinued the study (the reasons were as follows: use of a prohibited concomitant medication, development of an adverse event with a causal relationship with the study drug, and the investigator’s judgment based on the subject’s condition [*n* = 1, for each]), but these subjects were included in the efficacy analysis because they had such data. Consequently, 56 subjects (NPC-02 group: *n* = 30, Placebo group: *n* = 26) were included in the efficacy analysis, while all 57 subjects (NPC-02 group: *n* = 31, Placebo group: *n* = 26) were included in the safety analysis (Fig. 1).

**Fig. 2:**
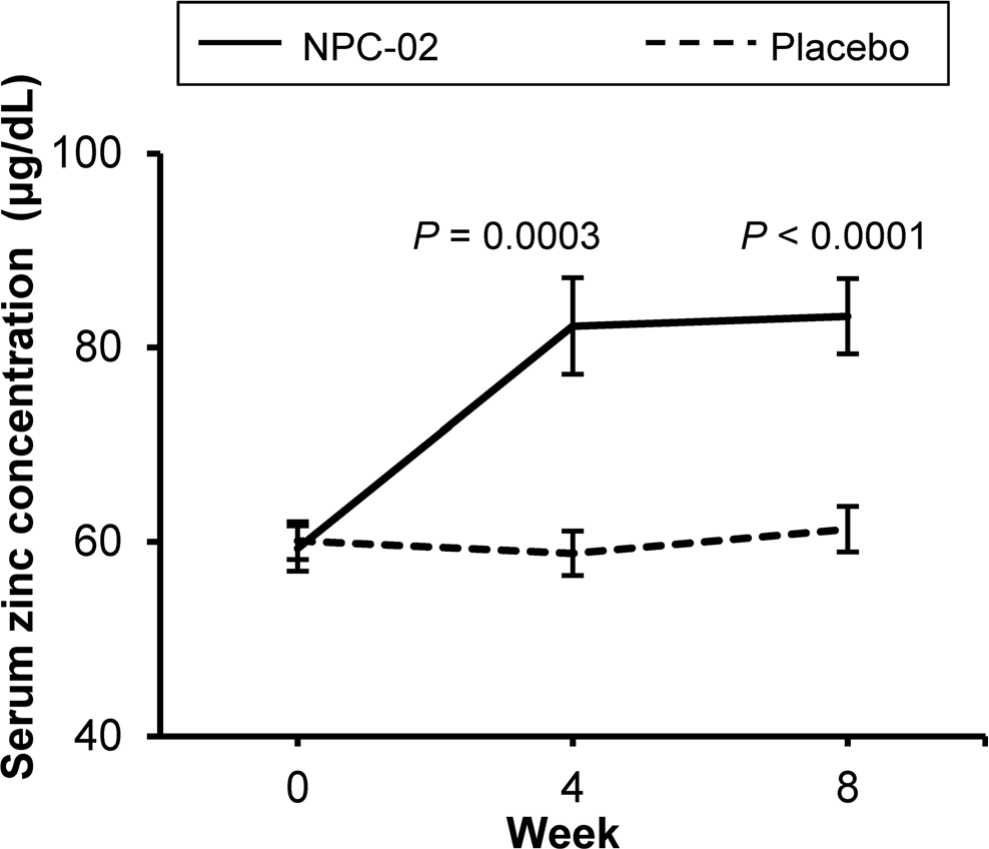
Changes in serum zinc concentrations in the NPC-02 and Placebo groups at weeks 4 and 8. The serum zinc concentration increment was larger in the NPC-02 group than in the Placebo group at weeks 4 and 8. NPC-02 group, NPC-02-treated group; Placebo group, placebo-treated group

Changes in the serum zinc concentration following drug administration for both groups are shown in Fig. 2. For the primary efficacy endpoint, the difference in serum zinc concentrations between the NPC-02 and Placebo groups was compared by applying analysis of covariance with the respective baseline values as the covariates. The serum zinc concentration showed a significant increase in the NPC-02 group as compared with the Placebo group at weeks 4 (*P* = 0.003) and 8 (*P* < 0.001).

Comparisons of changes in clinical laboratory parameters between the NPC-02 and Placebo groups following drug administration are shown in Table 2 and Table S1 (Online Resource 1). The serum zinc concentrations at weeks 4 and 8 were significantly higher in the NPC-02 group than in the Placebo group (*P* < 0.0001). Alkaline phosphatase (ALP), amylase, and lipase concentrations at week 4 were also higher in the NPC-02 group; however, no significant differences in these concentrations were found at week 8.

**Table 2:** Changes in main clinical laboratory parameters

Laboratory parameter	Evaluation time point (week)	NPC-02 group			Placebo group			*P* value^b^
		*n*	Actual value^*^	Change^a^	*n*	Actual value^a^	Change^a^	
Zinc (μg/dL)	0	31	58.6 ± 13.2	–	26	60.1 ± 9.9	–	–
	4	29	82.2 ± 26.8	22.7 ± 24.9	26	58.8 ± 11.7	− 1.3 ± 8.3	< 0.0001^*^
	8	27	83.2 ± 20.2	24.1 ± 16.6	26	61.3 ± 12.0	1.2 ± 6.4	< 0.0001^*^
ALP (U/L)	0	31	427.7 ± 264.0	–	26	467.3 ± 329.0	–	–
	4	29	465.5 ± 255.9	37.2 ± 71.9	26	416.4 ± 240.1	− 51.0 ± 120.9	0.0016^*^
	8	27	453.0 ± 268.3	23.1 ± 83.3	26	449.9 ± 271.8	− 17.4 ± 126.8	0.1736
Amylase (U/L)	0	31	93.3 ± 28.6	–	26	103.5 ± 37.8	–	–
	4	29	101.3 ± 32.0	5.6 ± 14.7	26	97.5 ± 34.2	− 6.0 ± 12.9	0.0032^*^
	8	27	97.4 ± 23.1	− 1.2 ± 17.5	26	102.2 ± 38.8	− 1.3 ± 13.3	0.9844
Lipase (U/L)	0	31	54.9 ± 21.3	–	26	57.2 ± 24.6	–	–
	4	29	58.8 ± 24.6	2.8 ± 10.2	26	53.7 ± 26.0	− 3.5 ± 10.6	0.0297^*^
	8	27	60.1 ± 23.9	3.1 ± 10.9	26	54.4 ± 24.4	− 2.8 ± 13.0	0.0803
Copper (μg/dL)	0	31	109.6 ± 22.6	–	26	116.9 ± 21.4	–	–
	4	29	112.0 ± 26.1	2.6 ± 11.3	26	116.5 ± 22.2	− 0.4 ± 9.6	0.3007
	8	27	109.2 ± 25.6	0.6 ± 12.4	26	117.5 ± 20.8	0.6 ± 7.5	0.9956

*ALP* alkaline phosphatase

*P < 0.05 was considered statistically significant

^a^Values are expressed as means ± SD

^b^Mann-Whitney test was used to statistically compare the changes from baseline between the two groups

The serum zinc concentration increment in the NPC-02 group at week 8 was 22.4 μg/dL greater than that in the Placebo group (*P* < 0.001) (Table 3).

**Table 3:** Changes in serum zinc concentration (FAS)

	Evaluation time point	Study group	Number of patients	Mean, μg/dL (standard deviation)	Least squares mean, μg/dL^a^ (95% confidence interval)	Difference in least squares mean, μg/dL (95% confidence interval)	*P* value
Change	Week 8^b^	NPC-02 group	30	23.7 (16.0)	23.7 (19.1–28.3)	22.4 (15.6–29.2)	< 0.001
		Placebo group	26	1.2 (6.4)	1.3 (−3.7–6.2)		

*FAS* full analysis set

^a^Analysis of covariance was performed with the baseline values as covariates

^b^At week 8, or at the time of drug discontinuation

In the analysis of the secondary endpoint in the subjects who completed treatment with the study drug, the proportions of subjects, whose serum zinc concentration had reached ≥ 80 μg/dL at weeks 4 and 8 in the NPC-02 and Placebo groups, were calculated (Table 4). The serum zinc concentration was ≥ 80 μg/dL in more than half of the subjects in the NPC-02 group at both evaluation time points (50.0% at both week 4 and week 8), whereas in the Placebo group, the serum zinc concentration reached ≥ 80 μg/dL at week 8 only in one subject (3.8%).

**Table 4:** Proportions of patients whose serum zinc concentration reached ≥ 80 μg/dL (FAS)

Evaluation time point	Study group	FAS	Number of patients (completed treatment with the study drug)^a^	< 80 μg/dL	≥ 80 μg/dL	Difference in proportion^b^ (95% confidence interval)
				Number of patients (%)	Number of patients (%)	
Week 4	NPC-02 group	30	29	14 (46.7)	15 (50.0)	50.00 (32.11–67.89)
	Placebo group	26	26	26 (100.0)	0 (0.0)	
Week 8	NPC-02 group	30	27	12 (40.0)	15 (50.0)	46.15 (26.80–65.51)
	Placebo group	26	26	25 (96.2)	1 (3.8)	

*FAS* full analysis set

^a^At week 4: one patient, who discontinued the drug due to an adverse event, was excluded. At week 8: two patients who discontinued the drug were excluded: one discontinued the study due to the use of a prohibited concomitant medication, and the other discontinued the study based on the investigator’s judgment

^b^Difference between the groups in the proportions of patients whose serum zinc concentrations was ≥ 80 μg/dL

The adverse drug reactions that occurred in both groups are listed in Table 5. Grade of events was estimated according to the National Cancer Institute Common Terminology Criteria for Adverse Events (version 4.0) [15]. No problematic adverse drug reactions were observed except for Grade 1 nausea and vomiting in one subject in the NPC-02 group, in whom the drug administration was discontinued based on the investigator’s judgment. This subject recovered after discontinuation of study drug administration.

**Table 5:** Adverse drug reactions in Study 1

Event	Number of patients	Grade of events^a^ (*n*)				Measures taken (*n*)	Recovery
		1	2	3	4		
Adverse drug reactions in NPC-02 group (*n* = 31)							
Nausea	2	2	0	0	0	No (1) Drug discontinuation (1)	Yes
Iron overload	1	1	0	0	0	No	Yes
Cough	1	1	0	0	0	No	Yes
Productive cough	1	1	0	0	0	No	Alleviated
Pruritus	2	2	0	0	0	No	Yes
Adverse drug reactions in Placebo group (*n* = 26)							
Purpura	1	0	1	0	0	No	Yes

^a^The grade was estimated according to the National Cancer Institute Common Terminology Criteria for Adverse Events (Version 4)

### Study 2: Dose Adjustment Study of NPC-02 in Patients with Zinc Deficiency

The baseline characteristics of subjects with and without liver diseases included in this study are listed in Table 6. There were significant differences in age and body weight between these two groups, probably due to the presence of children in the group of subjects without liver diseases. Also, the alanine aminotransferase (ALT), aspartate aminotransferase (AST), total bilirubin, and albumin concentrations, which differed between the two groups, reflected the inclusion of subjects with chronic liver disease in one of the two groups. There were also differences in the amylase, lipase, and creatinine concentrations between the two groups. One of the reasons for these differences may be that five of the subjects with chronic liver diseases had concomitant chronic pancreatitis, hyperlipasemia, or hyperamylasemia, and three of the subjects with chronic liver diseases had concomitant renal dysfunction, whereas none of the subjects without liver diseases had concomitant symptoms of pancreatic or renal diseases. However, there was essentially no difference in baseline serum zinc concentrations between the two groups.

**Table 6:** Baseline characteristics of subjects in Study 2

	Subjects with chronic liver diseases (*n* = 22)	Subjects without liver diseases (*n* = 21)	*P* value
Sex (M/F)	13/9	9/12	0.366^a^
Age (years)	68.0 ± 11.03	32.2 ± 26.91	< 0.0001^b*^
Body weight (kg)	64.0 ± 12.80	40.9 ± 17.98	< 0.0001^b*^
Primary disease	Chronic hepatitis/liver cirrhosis/hepatic dysfunction	Rheumatoid arthritis/skin disease/short stature/others	–
	4/18/0	6/6/6/3	
	Causes of liver disease (HBV/HCV/HBV + HCV/Alcohol/NAFL/PBC/NBNC/others)	NA	
	0/8/1/2/2/2/7/0		
ALT (U/L)	36.27 ± 33.42	21.90 ± 19.31	0.007^b*^
AST (U/L)	48.18 ± 28.18	29.76 ± 15.58	0.004^b*^
ALP (U/L)	434.36 ± 143.21	524.76 ± 402.35	0.618^b^
Total bilirubin (mg/dL)	1.18 ± 0.67	0.84 ± 1.19	0.002^b*^
Albumin (g/dL)	3.55 ± 0.43	4.08 ± 0.36	0.0002^b*^
Amylase (U/L)	112.05 ± 29.60	95.43 ± 30.00	0.040^b*^
Lipase (U/L)	46.36 ± 26.45	22.29 ± 7.84	< 0.0001^b*^
Creatinine (mg/dL)	0.79 ± 0.28	0.54 ± 0.20	0.002^b*^
Zinc (μg/dL)	55.75 ± 9.67	60.05 ± 6.38	0.189^b^

Values are expressed as means ± SD

*ALP* alkaline phosphatase, *ALT* alanine aminotransferase, *AST* aspartate aminotransferase, *HBV* hepatitis B virus, *HCV* hepatitis C virus, *NAFL* nonalcoholic fatty liver, *NBNC* non-hepatitis B virus and non-hepatitis C virus, *PBC* primary biliary cholangitis

*P < 0.05 was considered statistically significant

^a^Test used for analysis: Fisher’s exact probability test

^b^Test used for analysis: two-sample *t* test

Four subjects with chronic liver diseases (three who had difficulty with dose adjustments, and one who developed a serious adverse event without a causal relationship with the study drug) and one free of liver diseases (development of an adverse event [nausea] with a causal relationship with the study drug) discontinued the study.

**Fig. 3:**
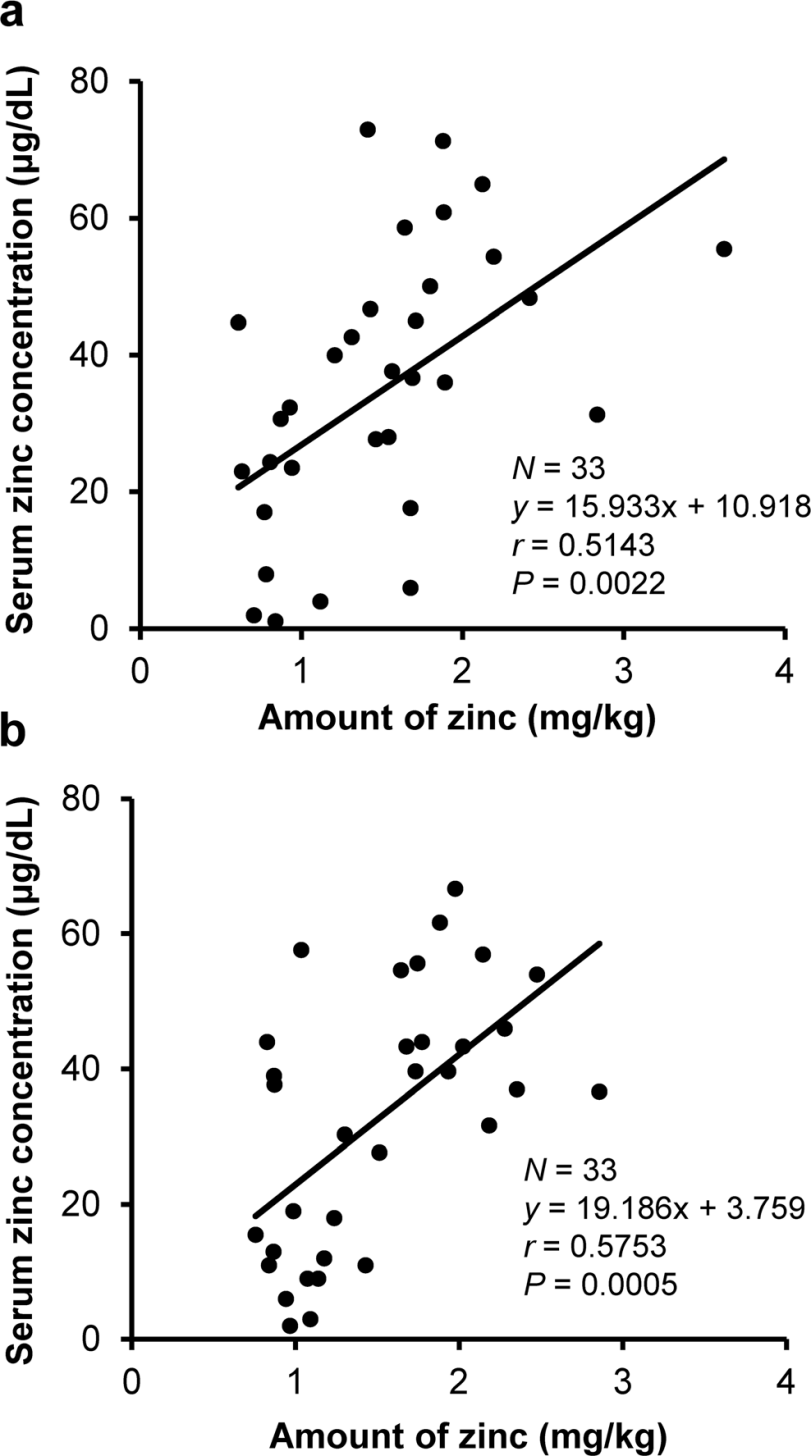
Increase in serum zinc concentration by amount of zinc from baseline in subjects with (**a**) and without (**b**) chronic liver diseases. Since this was a dose adjustment study, multiple data were available for one subject who had received increasing NPC-02 doses, and all of these data were included in the analysis. The serum zinc concentration was dose-dependently increased in subjects both with and without liver diseases

Because of the significant differences in body weight between the two groups, the serum zinc concentration increments from the start of administration were analyzed after dividing the prescribed doses for subjects by their body weights (mg/kg). The results demonstrated dose-dependent increases in the serum zinc concentration in both groups, i.e., those with and without liver diseases (Fig. 3a, b). One subject free of liver disease, who discontinued the study due to severe vomiting, was judged to have insufficient exposure to the drug, and therefore excluded from the analysis.

**Fig. 4:**
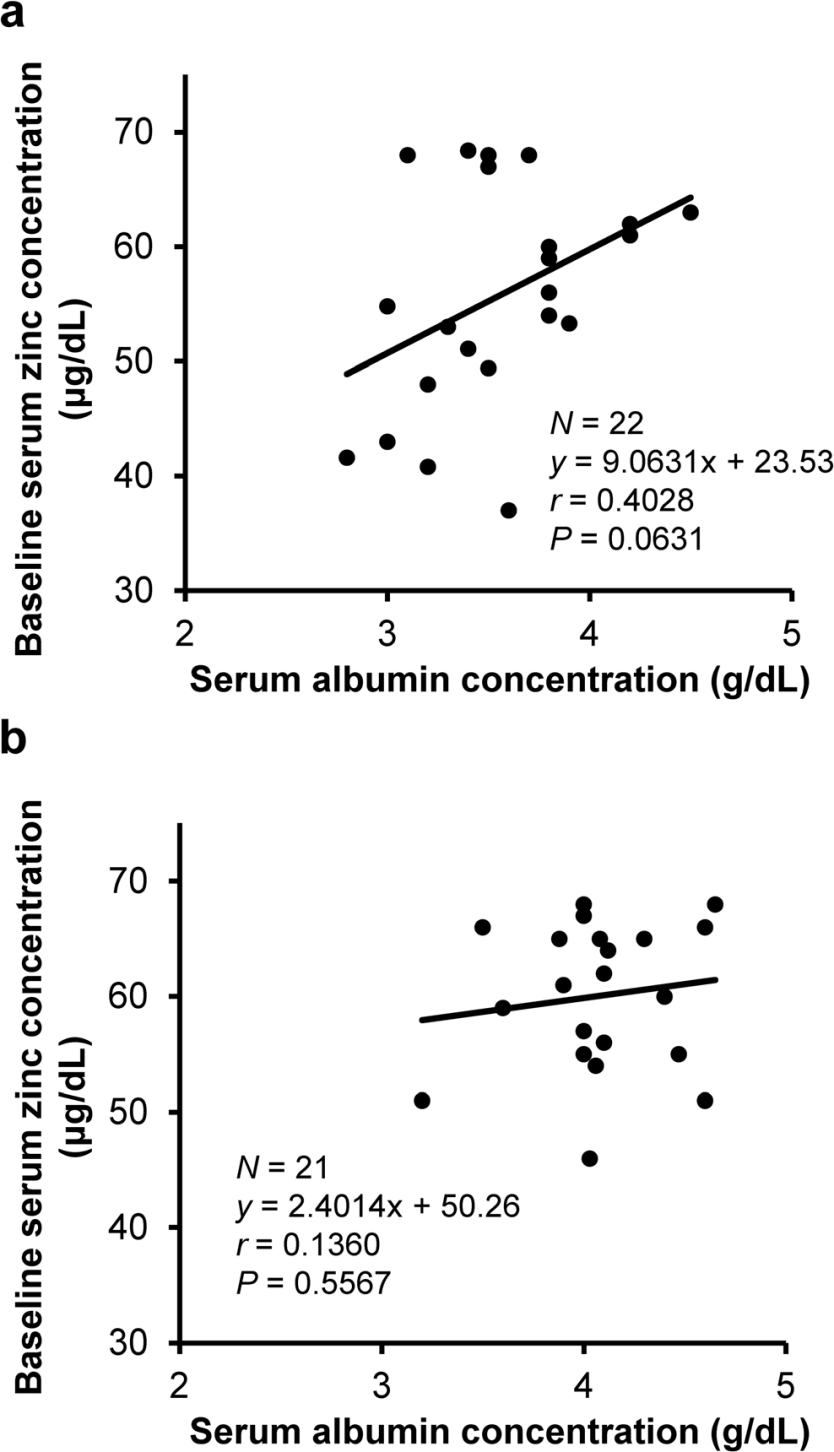
Correlation between baseline serum zinc and albumin concentrations in patients with (**a**) and without (**b**) chronic liver diseases. There was a correlation between baseline serum zinc and albumin concentrations, which did not reach statistical significance but was marginally positive (*P* = 0.063), in subjects with but not in those without liver diseases

The relationship between zinc and albumin concentrations at baseline was analyzed. There was a correlation between these concentrations, which did not reach statistical significance but was marginally positive (*P* = 0.063), in subjects with but not in those without chronic liver diseases (Fig. 4a, b).

The results demonstrated the number of subjects with zinc deficiency who maintained the target serum zinc concentration for 8 weeks (≥ 80 μg/dL but < 200 μg/dL) to be 37, a proportion of 86.0% (37/43). This proportion was considered to be high enough to demonstrate the reliability of the dose adjustment method used in this study, and the favorable maintenance effect of NPC-02 administration once the target serum concentration was reached.

The adverse drug reactions that occurred in this study are listed in Table 7. Lipase concentrations increased in seven subjects, while decreased copper concentrations and vomiting were noted in three subjects each. Additionally, Grade 3 (G3) ALP elevation occurred in one subject, and G3 lipase concentration elevation in three subjects. The case with an increased ALP concentration showed G2 at the start of drug administration, which was graded up to G3 at week 4, but zinc administration was continued despite this change. At week 8, the ALP elevation was still at G3, and drug administration was discontinued by the investigator due to the subject developing vomiting/nausea. As to those with an increased lipase concentration, one subject showed lipase concentration elevation after the completion of zinc administration, another showed an increase at one evaluation time point but recovered without drug discontinuation, and the third had G3 lipase elevation which predated the start of the study. In the third subject, lipase elevation was still G3 at week 4 of zinc administration, but improved despite continued NPC-02 administration.

**Table 7:** Adverse drug reactions in Study 2 (total number of subjects: 43)

Event	Number of subjects	Grade of events^a^ (*n*)				Measures taken (*n*)	Recovery
		1	2	3	4		
Abdominal pain (epigastric discomfort)	1	1	0	0	0	No	Yes
Diarrhea	1	1	0	0	0	No	Yes
Nausea	2	2	0	0	0	No (1) Drug discontinuation (1)	Yes
Pancreatitis	1	0	1	0	0	No	Yes
Vomiting	3	3	0	0	0	No (2) Drug discontinuation (1)	Yes
Alkaline phosphatase increased	2	1	0	1	0	No	Yes (1) Alleviated (1)
Lipase increased	7	3	1	3	0	No	Yes (5) Alleviated (2)
Amylase increased	1	1	0	0	0	No	Yes
Metabolism and nutrition disorders - Copper decreased	3	3	0	0	0	No	Yes (1) Alleviated (2)
Metabolism and nutrition disorders - Iron decreased	2	2	0	0	0	No	Yes
Metabolism and nutrition disorders - Ammonia increased	1	1	0	0	0	No	Yes
Palmar-plantar erythrodysesthesia syndrome	1	1	0	0	0	No	Yes

^a^The grade was estimated according to the National Cancer Institute Common Terminology Criteria for Adverse Events (Version 4)

We observed no other adverse drug reactions that were problematic for continuing the study. The subject with a decreased copper concentration showed resolution of this finding after zinc administration had been completed.

## Discussion

The findings of this study demonstrate that NPC-02 is effective for significant increase in the serum zinc concentration in hypozincemic patients with chronic liver diseases, and that it is dose-dependently effective in patients both with and without liver diseases. Whether or not pathological conditions presumably caused by zinc deficiency can also be ameliorated, by normalizing the patient’s zinc concentration with drug administration, is an important issue to be addressed in future studies.

The liver plays a central role in nutrient metabolism. Patients with liver cirrhosis have abnormalities in protein-energy metabolism, which worsen their outcomes and cause various complications including hepatic encephalopathy [16–18]. Previous studies demonstrated the efficacy of nutritional intervention in patients with liver cirrhosis accompanied by metabolic and nutritional disorders [19–21]. Other reports demonstrated that administration of branched-chain amino acids (BCAA) improves metabolic and nutritional disorders, resulting in reduced complications and the suppression of liver carcinogenesis, eventually improving outcomes, including survival [20–22]. Numerous distinct trace elements are present in the body, among which zinc was recently found to be intimately and crucially involved in the pathology of liver cirrhosis [3].

Zinc contributes to ammonia metabolism in liver diseases, and many reports have demonstrated zinc supplementation to impact ammonia metabolism improvement in patients with liver cirrhosis [3, 4, 10, 13, 23]. Hyperammonemia accelerates the consumption of BCAA during glutamine synthesis, which is the metabolic pathway of ammonia in skeletal muscles, causing a decrease in BCAA [22, 23], with BCAA deficiency in turn inducing an amino acid imbalance, which leads to a reduction in protein synthesis capacity [22, 23]. Thus, an improvement in ammonia metabolism, for example, that is induced by zinc supplementation, could potentially ameliorate not only hepatic encephalopathy but also overall protein metabolism.

Since our studies demonstrated that NPC-02 can improve hypozincemia in patients with liver cirrhosis, NPC-02 is expected to improve nitrogen metabolism with ammonia at the core of the associated mechanism. However, we could not examine the effects of NPC-02 on hyperammonemia in the present studies because only a small number of participants were hyperammonemic. Additionally, the amount of zinc (50 mg/day in Study 1, and a gradual increase from 50 to 150 mg/day in Study 2) was smaller, and the durations of administration (8 weeks in Study 1, and 8 weeks after reaching the target serum concentration [≥ 80 μg/dL but < 200 μg/dL] in Study 2) were shorter than those in previous studies of zinc supplementation therapy for hyperammonemia.

Many studies have examined the efficacy of zinc supplementation therapy [3, 10, 13]; however, only a few focused on a target serum zinc concentration. In our dose adjustment study, we set the target serum zinc concentration as ≥ 80 μg/dL. Pathological conditions possibly caused by zinc deficiency vary widely, and the serum zinc concentrations necessary to ameliorate each pathological state may also vary depending on the condition to be treated. In the reports demonstrating the efficacy of zinc supplementation for hyperammonemia, Katayama and colleagues [13] increased the mean baseline serum zinc concentration of 55 μg/dL to approximately 120 μg/dL. Marchesini and colleagues [10] similarly increased the serum zinc concentration by approximately 60%, from 53–84 μg/dL to approximately 100 μg/dL. Both studies documented an ammonia-lowering effect of the increased serum zinc concentration. A separate cohort study of patients with liver cirrhosis who had not developed cancer demonstrated that the survival rate without cancer development was significantly higher in patients who maintained their serum zinc concentrations at ≥ 80 μg/dL than in other patients [24]. Imai and colleagues reported that the recurrence and survival rates after surgery for hepatocellular carcinoma were significantly poorer in patients with a serum zinc concentration of ≤ 65 μg/dL than in those with a serum zinc concentration of > 65 μg/dL [25]. Zinc plays a variety of roles in immunocompetence, carcinogenesis, and fibrosis, and zinc deficiency in liver cirrhosis might thus be involved in many pathological conditions. Further research is warranted regarding the degrees to which zinc supplementation is effective in each of the zinc deficiency-associated pathological conditions.

We analyzed the relationships between the pre- and post-NPC-02 administration zinc and albumin concentrations, and found a marginally positive correlation between these concentrations in patients with but not in those without chronic liver diseases. These findings indicate that zinc is involved in the decreases in albumin concentrations observed in chronic liver diseases, which is consistent with previous reports that zinc is involved in nitrogen metabolism in liver diseases [3, 4, 10, 13]. The findings also suggest that zinc is unlikely to contribute to decreased albumin concentrations in patients without liver diseases. The mechanisms behind these effects should be addressed in future studies.

Concerning the adverse events in our studies, only one subject each in studies 1 and 2 discontinued NPC-02 administration, both due to nausea (one also had vomiting). There were no other problematic adverse events. NPC-02 has been used as a therapeutic agent for Wilson’s disease, and studies conducted during its development phases found no serious problems following its administration. However, physicians should pay close attention to the blood copper concentration, as well as to the serum zinc concentration, during any future administration of NPC-02, because NPC-02 supplementation is reportedly associated with decreased blood copper concentrations [26, 27]. Zinc reportedly may have immunosuppressive effects and reduce leukocyte chemotaxis [28]. However, one study found no adverse effects on lymphocyte function [29]. Although, in our study, no clinically relevant immunosuppressive effects of zinc were observed, we consider it necessary to clarify the effects of chronic high-dose zinc administration on immune functions in a future study.

This study has limitations. Although we investigated the effects of NPC-02 on serum zinc concentrations, clinical indexes were not a focus of this study. Many reports have confirmed that clinical indexes, including hyperammonemia, correlate with serum concentrations [3–10]. Nonetheless, only a few RCT have shown the relationship between the administration of a zinc formulation and the serum zinc concentration [13]. Investigation into the effect of administering a zinc formulation on clinical indexes is, thus, an issue to be addressed in the future.

## Conclusions

The zinc acetate formulation NPC-02, used in our current studies, dose-dependently increased the serum zinc concentration. Therefore, NPC-02 was suggested to be an effective therapy in patients with liver cirrhosis, in which frequent and severe zinc deficiency is observed.

## Electronic Supplementary Material

ESM 1 (PDF 186 kb)
