# Supplementary material for: Effects of Zinc Acetate on Serum Zinc Concentrations in Chronic Liver Diseases: a Multicenter, Double-Blind, Randomized, Placebo-Controlled Trial and a Dose Adjustment Trial
Source: Biol Trace Elem Res. 2019 Aug 7;195(1):71–81. doi: 10.1007/s12011-019-01851-y (PMC7150658; doi:10.1007/s12011-019-01851-y)
Supplement: Supplementary file 1 — (PDF 186 kb) [file 12011_2019_1851_MOESM1_ESM.pdf]

Article title: Effects of zinc acetate on serum zinc concentrations in chronic liver diseases: A multicenter, double-blind, randomized, placebo-controlled trial and a dose adjustment trial

Journal name: Biological Trace Element Research

Author names: Kazuhiro Katayama*, Atsushi Hosui, Yoshiyuki Sakai, Minoru Itou, Yasushi Matsuzaki, Yoriyuki Takamori, Keiko Hosho, Tomomi Tsuru, Yasuhiro Takikawa, Kojiro Michitaka, Eishin Ogawa, Yoko Miyoshi, Toshifumi Ito, Shinobu Ida, Izumi Hamada, Katsunori Miyoshi, Hiroko Kodama, Tetsuo Takehara

*__Corresponding author:__ Kazuhiro Katayama

Affiliation: Department of Hepato-biliary and Pancreatic Oncology, Osaka International Cancer Institute, Osaka, Japan

E-mail address: katayama-ka@mc.pref.osaka.jp

Supplementary material

Table S1 Changes in clinical laboratory parameters

Laboratory parameter	Evaluation time point (week)	NPC-02 group			Placebo group			*P* value[b]
		n	Actual value[a]	Change[a]	*n*	Actual value[a]	Change[a]	
WBC (μL^{-1})	0	31	4435.5 ± 1388.7	—	26	4280.8 ± 1497.7	—	—
	4	29	4458.6 ± 1327.6	−37.9 ± 622.4	26	4253.8 ± 1486.0	−26.9 ± 633.4	0.9485
	8	27	4466.7 ± 1538.0	133.3 ± 1129.7	26	4334.6 ± 1466.7	53.8 ± 741.5	0.7642
Hemoglobin (g/dL)	0	31	12.86 ± 1.83	—	26	12.59 ± 1.53	—	—
	4	29	12.95 ± 1.81	0.03 ± 0.55	26	12.63 ± 1.49	0.04 ± 0.68	0.9481
	8	27	12.82 ± 1.63	0.01 ± 0.66	26	12.77 ± 1.47	0.18 ± 0.89	0.4229
Plt (10^{10}/L)	0	31	13.45 ± 7.64	—	26	12.64 ± 5.00	—	—
	4	29	13.30 ± 6.12	0.32 ± 1.97	26	12.45 ± 5.44	−0.19 ± 1.53	0.2874
	8	27	13.05 ± 6.86	0.46 ± 1.39	26	12.64 ± 6.12	0.00 ± 1.89	0.3168

Laboratory parameter	Evaluation time point (week)	NPC-02 group			Placebo group			*P* value[b]
		n	Actual value[a]	Change[a]	*n*	Actual value[a]	Change[a]	
Reticulocyte (Permil)	0	31	16.9 ± 6.5	−	26	18.5 ± 6.9	−	−
	4	29	16.2 ± 5.7	−0.7 ± 2.8	26	16.8 ± 6.5	−1.7 ± 5.0	0.3403
	8	27	16.3 ± 6.0	−0.9 ± 2.7	26	17.5 ± 7.1	−1.0 ± 4.2	0.9098
ALT (U/L)	0	31	31.5 ± 14.2	−	26	47.0 ± 39.6	−	−
	4	29	32.4 ± 17.4	0.7 ± 7.6	26	44.2 ± 40.5	−2.8 ± 15.2	0.2828
	8	27	31.8 ± 16.5	0.5 ± 6.6	26	43.2 ± 29.9	−3.7 ± 17.7	0.2534
AST (U/L)	0	31	44.4 ± 19.2	−	26	51.1 ± 27.4	−	−
	4	29	42.4 ± 16.8	−2.2 ± 6.7	26	51.1 ± 31.8	0.0 ± 16.5	0.5234
	8	27	42.3 ± 18.7	−2.8 ± 7.5	26	49.9 ± 23.1	−1.2 ± 16.4	0.6515
ALP (U/L)	0	31	427.7 ± 264.0	−	26	467.3 ± 329.0	−	−
	4	29	465.5 ± 255.9	37.2 ± 71.9	26	416.4 ± 240.1	−51.0 ± 120.9	0.0016*
	8	27	453.0 ± 268.3	23.1 ± 83.3	26	449.9 ± 271.8	−17.4 ± 126.8	0.1736

Laboratory parameter	Evaluation time point (week)	NPC-02 group			Placebo group			P value[b]
		n	Actual value[a]	Change[a]	n	Actual value[a]	Change[a]	
γ-GTP (U/L)	0	31	52.3 ± 82.6	—	26	99.2 ± 243.7	—	—
	4	29	55.7 ± 83.7	0.6 ± 8.1	26	75.0 ± 107.7	−24.2 ± 143.8	0.3582
	8	27	50.8 ± 72.1	−4.0 ± 19.3	26	113.0 ± 303.2	13.8 ± 60.4	0.1506
Total bilirubin (mg/dL)	0	31	1.09 ± 0.66	—	26	0.84 ± 0.35	—	—
	4	29	1.06 ± 0.64	−0.06 ± 0.42	26	0.85 ± 0.36	0.01 ± 0.21	0.4676
	8	27	1.07 ± 0.58	−0.07 ± 0.27	26	0.94 ± 0.49	0.10 ± 0.38	0.0665
Albumin (g/dL)	0	31	3.65 ± 0.46	—	26	3.79 ± 0.50	—	—
	4	29	3.68 ± 0.39	0.01 ± 0.15	26	3.76 ± 0.52	−0.03 ± 0.18	0.3536
	8	27	3.69 ± 0.43	0.03 ± 0.22	26	3.77 ± 0.52	−0.02 ± 0.17	0.3289
Amylase (U/L)	0	31	93.3 ± 28.6	—	26	103.5 ± 37.8	—	—
	4	29	101.3 ± 32.0	5.6 ± 14.7	26	97.5 ± 34.2	−6.0 ± 12.9	0.0032*
	8	27	97.4 ± 23.1	−1.2 ± 17.5	26	102.2 ± 38.8	−1.3 ± 13.3	0.9844

Laboratory parameter	Evaluation time point (week)	NPC-02 group			Placebo group			P value[b]
		n	Actual value[a]	Change[a]	*n*	Actual value[a]	Change[a]	
Lipase (U/L)	0	31	54.9 ± 21.3	–	26	57.2 ± 24.6	–	–
	4	29	58.8 ± 24.6	2.8 ± 10.2	26	53.7 ± 26.0	−3.5 ± 10.6	0.0297*
	8	27	60.1 ± 23.9	3.1 ± 10.9	26	54.4 ± 24.4	−2.8 ± 13.0	0.0803
Total cholesterol (mg/dL)	0	31	145.1 ± 35.0	–	26	156.4 ± 26.5	–	–
	4	29	147.6 ± 32.2	1.0 ± 12.7	26	156.0 ± 31.6	−0.4 ± 14.1	0.6961
	8	27	144.1 ± 31.5	−1.3 ± 13.6	26	154.5 ± 24.8	−1.9 ± 12.7	0.8634
Triglyceride (mg/dL)	0	31	89.4 ± 50.2	–	26	95.6 ± 42.8	–	–
	4	29	85.1 ± 41.9	−5.4 ± 28.1	26	96.4 ± 43.8	0.8 ± 25.0	0.3891
	8	27	93.9 ± 56.7	5.6 ± 37.7	26	102.6 ± 60.4	7.0 ± 39.7	0.8951
BUN (mg/dL)	0	31	14.88 ± 4.74	–	26	15.55 ± 5.59	–	–
	4	29	16.86 ± 5.45	1.51 ± 2.69	26	15.08 ± 7.21	−0.47 ± 5.04	0.0707
	8	27	15.90 ± 4.43	0.91 ± 2.79	26	15.32 ± 5.16	−0.22 ± 3.36	0.1878

Laboratory parameter	Evaluation time point (week)	NPC-02 group			Placebo group			*P* value[b]
		n	Actual value[a]	Change[a]	*n*	Actual value[a]	Change[a]	
Creatinine (mg/dL)	0	31	0.763 ± 0.218	−	26	0.685 ± 0.205	−	−
	4	29	0.798 ± 0.210	0.012 ± 0.096	26	0.677 ± 0.200	−0.008 ± 0.054	0.3480
	8	27	0.744 ± 0.168	−0.034 ± 0.076	26	0.681 ± 0.180	−0.004 ± 0.105	0.2397
Iron (μg/dL)	0	31	96.5 ± 55.0	−	26	106.8 ± 48.8	−	−
	4	29	98.7 ± 54.4	−2.0 ± 57.1	26	117.2 ± 68.5	10.4 ± 44.4	0.3749
	8	27	99.6 ± 48.7	0.8 ± 42.3	26	133.1 ± 76.0	26.3 ± 59.9	0.0778
Copper (μg/dL)	0	31	109.6 ± 22.6	−	26	116.9 ± 21.4	−	−
	4	29	112.0 ± 26.1	2.6 ± 11.3	26	116.5 ± 22.2	−0.4 ± 9.6	0.3007
	8	27	109.2 ± 25.6	0.6 ± 12.4	26	117.5 ± 20.8	0.6 ± 7.5	0.9956
Zinc (μg/dL)	0	31	58.6 ± 13.2	−	26	60.1 ± 9.9	−	−
	4	29	82.2 ± 26.8	22.7 ± 24.9	26	58.8 ± 11.7	−1.3 ± 8.3	<0.0001*
	8	27	83.2 ± 20.2	24.1 ± 16.6	26	61.3 ± 12.0	1.2 ± 6.4	<0.0001*

[a]Values are expressed as means ± SD.

[b]Mann-Whitney test was used to statistically compare the changes from baseline between the two groups.

[*]$P < 0.05$ was considered statistically significant.

ALP, alkaline phosphatase; ALT, alanine aminotransferase; AST, aspartate aminotransferase; BUN, blood urea nitrogen; γ-GTP, γ-glutamyl transpeptidase; Plt, platelet count; WBC, white blood cell count.
